# Epigenetic signatures in surrogate tissues are able to assess cancer risk and indicate the efficacy of preventive measures

**DOI:** 10.1038/s43856-025-00779-w

**Published:** 2025-04-02

**Authors:** James E. Barrett, Chiara Maria Herzog, Sepideh Aminzadeh-Gohari, Elisa Redl, Isma Ishaq Parveen, Julia Rothärmel, Julia Tevini, Daniela D. Weber, Luca Catalano, Victoria E. Stefan, Thomas K. Felder, Peter Obrist, Twana Alkasalias, Kristina Gemzell-Danielsson, Roland Lang, Barbara Kofler, Martin Widschwendter

**Affiliations:** 1 European Translational Oncology Prevention and Screening (EUTOPS) Institute, University Innsbruck, Hall in Tirol, Austria; 2 Institute for Biomedical Aging Research, University Innsbruck, Innsbruck, Austria; 3 Research Program for Receptor Biochemistry and Tumor Metabolism, Department of Pediatrics, University Hospital of the Paracelsus Medical University, Salzburg, Austria; 4 Department of Bioscienes and Medical Biology, University of Salzburg, Salzburg, Austria; 5 Department of Laboratory Medicine, University Hospital of the Paracelsus Medical University, Salzburg, Austria; 6 Institute of Pharmacy, Paracelsus Medical University, Salzburg, Austria; 7 Tyrolpath Obrist Brunhuber GmbH, Zams, Austria; 8 General Directorate of Scientific Research Center, Salahaddin University-Erbil, Erbil, 44001 Iraq; 9 Department of Women’s and Children’s Health, Karolinska Institutet and Karolinska University Hospital, Stockholm, Sweden; 10 Department of Dermatology and Allergology, University Hospital of the Paracelsus Medical University, Salzburg, Austria; 11 Department of Women’s Cancer, University College London, London, UK

**Keywords:** Breast cancer, Cancer prevention

## Abstract

**Background:**

In order to advance personalized primary cancer prevention, surrogate endpoint biomarkers in distant, easy to access tissues (i.e., field defect indicators) reflecting field cancerization in the organ at risk are essential.

**Methods:**

Here we utilized medroxyprogesterone acetate and 7,12-dimethylbenzanthracene to induce mammary gland cancers in mice. We assessed epigenetic signatures reflective of carcinogen exposure, cell-type composition, mitotic age, and methylation at progesterone receptor binding sites in both, the tissue at risk (normal mammary gland; field cancerization) and distant non-at-risk organs (cervix, oviduct, and blood; field defect indicators), in mice that did and did not develop mammary gland cancers.

**Results:**

We demonstrate that the anti-progestine mifepristone reduces the cancer risk by more than 50%. Importantly, the reduction in cancer risk is accompanied by a decline in both field cancerization and field defect indicators; specifically, epigenetic signatures in the cervix are predictive of mammary cancer formation but show tissue-specific directionality.

**Conclusions:**

These data encourage further exploration of epigenetic biomarkers in certain field defect-indicating tissues with a view to monitor the efficacy of cancer prevention strategies in humans.

## Abstract (web summary)

Barrett, Herzog, and Aminzadeh-Gohari et al. demonstrate that tissue-specific epigenetic DNA methylation signatures define field cancerization and can predict cancer formation, even from distant sources. Mifepristone reduced mammary gland cancer risk in mice by over 50%, with this effect mirrored in changes to cervical DNA methylation patterns.

## Plain Language Summary

Molecules within the body that can be used as indicators that cancer is present are called biomarkers. Ideally, they are obtained from parts of the body that are easy to access, such as the blood, even if they are far from the actual site of cancer. Here, we used mice to model breast cancer and looked at biomarkers in the mammary gland, where cancer occurs, distant organs (including the cervix) and blood. We found that the amount of the hormone progesterone present has a role in increasing cancer risk. Mifepristone, an existing drug that blocks the effects of progesterone, reduced the risk of mammary gland cancer by over 50%. Changes in biomarkers in the cervix could be used to predict whether the mouse would develop cancer in the mammary gland, suggesting that these markers could help monitor whether cancer prevention strategies are working in humans.

## Introduction

Cancer is likely to become the most prevalent disease and the most common cause of death in the near future^1^. Currently, primary cancer prevention strategies are still limited. It is well known that specific risk factors, including progesterone exposure, drive a phenomenon called field cancerization, which is the replacement of the normal cell population by a cancer-primed cell population that shows no morphological changes^2^. Whereas field cancerization by definition is limited to the organ at risk (e.g., breast^3^ or fallopian tube^4^), preliminary evidence supports the idea that field defect indicators exist in non-at-risk organs (e.g., blood or cervical cells) distant from the tissue at risk. Therefore, field defect-indicating biomarkers are suitable to assess the cancer risk^5,6^. There is no evidence yet whether these field defects could also reflect the efficacy of cancer preventive measures.

Breast cancer is by far the most common cancer in women and among the three most common cancers in general^7^. High (e.g., due to a germline *BRCA1* mutation^8^) and/or prolonged (e.g., hormone replacement therapy^9,10^) exposure to the sex steroid hormone progesterone, impacting receptor activator of nuclear factor-kB (RANK), wingless/integrated (WNT), and other signaling pathways, is a key driver for the development of breast cancers with the worst prognoses^11–15^. Preliminary evidence exists that progesterone also drives ovarian carcinogenesis^16^.

Progesterone antagonism could represent a promising strategy for the prevention of breast cancers with the worst prognosis. Indeed, there is preliminary evidence that the progesterone receptor modulator mifepristone may reduce *BRCA1*-mediated mammary gland cancer in mice^17^. In humans, data from a small exploratory study showed that mifepristone exposure reduced levels of markers associated with high cancer risk (i.e., mitotic age and the proportion of luminal progenitor cells) in normal breast tissue^18^.

Currently, surgical intervention such as double mastectomy is still the only effective cancer-risk reduction strategy for women at the highest risk^19^. Utilization of mifepristone for primary cancer prevention has been hampered by two independent factors: (a) due to its pregnancy-terminating property, access to mifepristone is extremely restricted in many US states, adding to the ethical dilemmas a ban on abortion generates^20^, and (b) a reliable and convenient (non-invasive) option to monitor cancer-preventive efficacy (i.e., validated surrogate endpoint biomarkers) is not currently available.

**Fig. 1:**
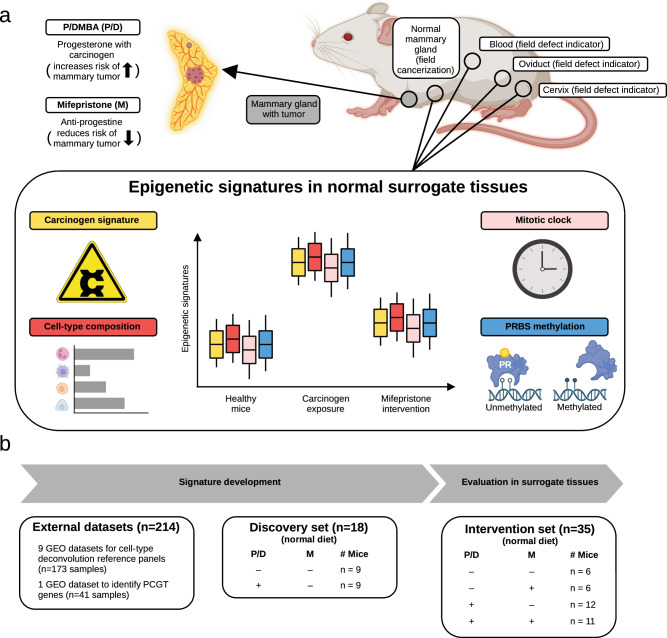
Overview of study design and datasets. **a** Overview of experimental design. **b** Ten GEO datasets were used to develop reference panels for cell-type deconvolution of bulk tissue samples and a mitotic clock. The Discovery set was used to develop a carcinogen signature. P/D, medroxyprogesterone acetate and 7,12-dimethylbenzanthracene; M, mifepristone; PR: progesterone receptor. Created with Biorender.com.

In this study, we used a chemical-induced and immune-competent mouse model to test whether (1) the carcinogen treatment drives epigenetic field cancerization in the organ at risk (i.e., changes observed in unaffected mammary glands) that is also detectable in distant field defect-indicating organs (i.e., cervix, oviduct, and blood) and (2) whether the efficacy of mifepristone to prevent mammary gland cancer can be assessed by epigenetic signatures in both the tissue at risk and field defect-indicating distant organs (Fig. 1a). We studied four epigenetic signatures reflecting different aspects of carcinogenesis and cancer prevention: (i) a signature reflecting carcinogen exposure; (ii) a signature indicating cell-type composition^18^; (iii) a mitotic clock based on methylation of polycomb group target (PCGT) genes^18^; and (iv) a signature indicating methylation at progesterone receptor binding sites (PRBS)^5^ (Fig. 1a). PRBS show significantly reduced DNA methylation (DNAme) in normal breast tissue from women with *BRCA1* mutation (Supplementary Fig. 1a^5^—provided again for comparison). These reduced methylation levels in PRBS can be restored by 2 months of mifepristone exposure^18^ (Supplementary Fig. 1b). This observation is consistent with the idea that frequent binding of transcription factors prevents DNAme^21,22^ but that the presence of an antagonist leads to underutilization, which facilitates DNAme and may make these cells less susceptible to progesterone signaling.

## Methods

### In vivo studies

Animal experiments were performed at the animal facility of the Paracelsus Medical University Salzburg in accordance with the Austrian federal ministry of education, science, and research (BMBWF), study approval No. 2021–0.236.530.

Mammary tumors were induced in 7 week old female BALB/c mice (Charles River) by subcutaneous implantation with 50 mg slow-release (90 days) medroxyprogesterone (P) pellets (cat #NP-161, Innovative Research of America) and oral gavage administration of 1 mg of 7,12-dimethylbenz[a]anthracene (D, cat #D3254, Sigma-Aldrich) dissolved in corn oil (cat # C8267, Sigma-Aldrich) once a week for a total of four to five times. On the same day of P pellet implantation, mice also received either placebo or 3 mg mifepristone (cat #M8046, Sigma-Aldrich) in the form of slow-release (90 days) pellets (cat #NX-999, Innovative Research of America) by subcutaneous implantation. In the same manner, mice without mammary tumor induction received placebo pellets (cat #NC-111, Innovative Research of America) and oral gavage containing corn oil as the corresponding vehicle.

Mice had free access to food and water. During the study tumor incidence, tumor size, and body weight were measured three times per week for mice with tumors. Otherwise, body weight was measured once per week. The tumor size was measured using a caliper and calculated using the formula: width × height × length / 2. Mice were euthanized when they reached 23 weeks of age or earlier if the size of mammary tumors exceeded 200 mm^3^. Blood was collected by cardiac puncture and stored in heparin-coated tubes on ice to first collect plasma and then peripheral blood mononuclear cells (PBMCs). All samples, including breast tumors and organs, plasma and PBMCs, were frozen in liquid nitrogen and then stored at −80 °C for DNAme analysis and LC-MS/MS quantification of mifepristone and metapristone. A piece of breast tumor and breast tissue were fixed in formalin and embedded in paraffin for histological analysis. All tumors were confirmed histologically by a pathologist after hematoxylin and eosin (HE) staining.

### Survival analysis

For Kaplan-Meier curves and fitting Cox proportional hazards models, an event was defined as the detection of a tumor (i.e., the tumor was measured with calipers). Mice that were sacrificed at 23 weeks or that were euthanized due to a loss of body weight without the detection of a tumor were regarded as censored. The time to event was calculated as the number of days from the date of implantation of either P/D, mifepristone, or placebo pellets to the date a tumor was first measured. Any tumor that was detected and confirmed histologically after sacrifice but had not been detected and measured before sacrifice was not regarded as an event in tumor-free survival analysis.

### Mifepristone and metapristone quantification

After sample preparation as outlined in ref. ^23^, a validated HPLC-MS/MS was used to quantify mifepristone and metapristone in plasma and organ samples^24^.

In brief, sample preparation consisted of a liquid-liquid extraction protocol. For this purpose, 2 μl of levonorgestrel (1 µg/ml) was added as an internal standard to 100 μl of plasma (or reconstituted tissue extracts) and vortexed for one minute before the addition of 900 µl diethylether. All samples were then centrifuged at 21,000 × *g* for 10 min at 4 °C. The supernatants were dried at room temperature under continuous nitrogen flow and reconstituted in 50 µl 20/80 (vol/vol) acetonitrile/water containing 0.1% formic acid.

Using an Ultra-Turrax (IKA), tissues were homogenized with ice-cold methanol. For mammary glands, mammary tumor, liver, and spleen tissues 6 µl solvent per 1 mg tissue were used, and uterus samples were homogenized in 12 µl solvent per 1 mg tissue. After homogenization, tissue homogenates were centrifuged at 2600 × *g* for 10 min at 4 °C, followed by another centrifugation step of the obtained supernatant at 14,000 × *g* for 10 min at 4 °C. The final tissue extracts were then dried at room temperature under a constant nitrogen flow and reconstituted in 100 µl diethylether. Mifepristone and metapristone extraction from tissue samples was then performed as described above.

Chromatographic separation was carried out on an ExionLC system (Sciex) using a Phenomenex Synergi Fusion-RP column (50 × 2 mm, 4 µm particle size, 80 Å pore size) operated at a temperature of 35 °C with water containing 0.1% formic acid as mobile phase A and 95/5 (vol/vol) acetonitrile/water containing 0.1% formic acid as mobile phase B. Gradient elution at a flow rate of 0.4 ml/min started from 20.0 to 80.0% B in 4.5 min, followed by a flushing step with 95.0% B for 1.0 min followed by a re-equilibration step with 20.0% B for 1.5 min. The total time for a single chromatographic run was 8.0 min. For plasma and tissue extracts, injection volumes of 15 µl were used.

Selected reaction monitoring (SRM) measurements for mifepristone and metapristone as well as for the internal standard levonorgestrel in the obtained samples were performed on a TripleQuad5500+ (Sciex) in positive ionization mode. The quantifier ion transitions were m/z 430.2 to 372.2 for mifepristone, m/z 416.2 to 358.3 for metapristone and m/z 313.1 to 245.2 for levonorgestrel. The method was fully validated according to the Eurachem Guideline^25^. Calibration curves were derived from ratios of the peak areas of mifepristone or metapristone and the internal standard using 1/χ-weighted linear least-squares regression of the area ratio versus the concentration of the corresponding internal standard. Analyst software 1.7.1 was used for acquisition, analysis, and quantification of data.

### Sample processing and DNA extraction

Blood cell and organ samples were stored at −80 °C until DNA extraction and DNA methylation analysis. DNA was isolated from cells and different organs using AllPrep DNA/RNA Mini Kits (cat #80204, Qiagen Ltd), following the manufacturer’s protocol. For organ samples, a maximum of 30 mg of tissue was used for DNA extraction. Tissue samples were disrupted in 600 µl RLT buffer using a pestle and mortar and homogenized using the QIAshredder homogenizer (cat #79656, Qiagen Ltd). DNA concentration was measured using the QuantiFluor® ONE dsDNA system (cat #E4870, Promega) on a SpectraMax® M2 microplate reader (Molecular Devices).

### DNA methylation array analysis

DNA concentration was normalized to 12.5 ng/µl in nuclease-free water, and 250 ng total DNA was bisulfite-modified using the EZ-96 DNA Methylation-Lightning Automation kit (#D5049, Zymo Research Corp, cat) on a Tecan Fluent® 480 liquid handling platform. Bisulfite modified DNA was eluted in 15 µl of nuclease-free water, and 8 µl of modified DNA were subjected to methylation analysis on the Illumina Infinium Mouse Methylation BeadChip microarray (Illumina, CA, USA) at the EUTOPS Institute according to the manufacturer’s standard protocol. Processed BeadChips were imaged within 24 h on the Illumina iScan^TM^-System.

### Statistics and reproducibility

All statistical analysis were performed in R version 4.4.1. A discovery set of 18 mice was used to develop the carcinogen signature (custom script provided)^26^. The mitotic clock and PRBS mean methylation quantities, and methods to infer the proportions of different cell types were also developed using external datasets. An independent validation set of 35 mice was used to validate these quantities.

### Methylation analysis

Methylation analysis was conducted using a custom pipeline (eutopsQC). Briefly, raw methylation microarray data were loaded using the R package minfi, version 1.46, with minor modifications to allow for annotation of the mouse array, which is not by default included in the minfi package. For this, we generated annotation (10.5281/zenodo.12721773) and manifest (10.5281/zenodo.12721813) R packages based on the array information provided by Illumina. Any samples with median methylated and unmethylated intensities <9.5 were removed. Any probes with a detection *p* value > 0.01 were regarded as failed. Any samples with >10% failed probes, and any probes with a >10% failure rate were removed from the dataset. A total of six samples were removed (one oviduct and two spleen samples from healthy mice in the Discovery set, and one mammary gland and two spleen samples from P/D-exposed mice in the Discovery set).

Background intensity correction and dye bias correction was performed using the minfi single sample preprocessNoob function. Probe bias correction was performed using the beta mixture quantile normalization (BMIQ) algorithm in the ChAMP package, version 2.30.0, with a modification to allow for processing of the mouse array. The top 1000 most variable probes (ranked by standard deviation) were used in a principal component analysis. Correlation or Kruskal-Wallis tests were performed in order to identify any anomalous associations between plate, sentrix position, date of array processing, date of DNA isolation, sampling date, DNA concentration, and the top ten principal components. No issues were identified.

### Carcinogen signature

For each of the nine tissue types in the discovery set, CpG sites were ranked according to the difference in mean beta values between the P/D-exposure group and the non-exposure group. A geometric mean was used to combine tissue-specific CpG rankings into a single pan-tissue ranking. A carcinogen signature was defined as the mean beta value across the top 1000 pan-tissue hyper-methylated CpGs minus the mean beta value across the top 1000 pan-tissue hypo-methylated CpGs. The signature was linearly scaled to have zero mean and unit standard deviation in healthy mammary gland samples from the discovery set.

### Inference of cell-type composition

The relative proportions of different cell subtypes in each sample were estimated using the EpiDISH package, version 2.16. A primary reference panel of cell-type specific CpGs for epithelial, fibroblast, fat, and immune cells was constructed for use with the EpiDISH algorithm. A total of 46 epithelial samples from 3 different GEO datasets, 7 fibroblast samples from 2 GEO datasets, 5 fat samples from 2 GEO datasets, and 118 immune cell samples from 3 GEO datasets were used to construct the reference panel (Supplementary Data 1).

These datasets were generated using a combination of the Illumina mouse methylation array, whole genome bisulfite sequencing (WGBS), and reduced representation bisulfite sequencing (RRBS). For the WGBS and RRBS datasets, beta values were calculated as the proportion of methylated reads divided by the total reads at a given CpG. Only CpGs that corresponded to CpG sites covered by the methylation array were considered. WGBS and RRBS datasets typically correspond to approximately 95% and 8% of CpGs on the array, respectively. CpGs that were missing from RRBS datasets or missing from more than 10% of samples were excluded from the analysis. This resulted in 15,290 CpGs for the remaining analysis.

Array based beta values followed a characteristic bimodal distribution with modes located at 0.017 and 0.955. Sequencing beta values also followed a bimodal distribution with modes located at 0 and 1 exactly, since there is no noise from background intensities. The following linear transformation was applied to sequencing beta values, beta_adjusted = 0.938*beta + 0.017, where beta is the original beta value. This rescales the sequencing beta values such that both array and sequencing distributions have equal modes.

For each CpG, the difference between the mean methylation level in epithelial cells and the mean methylation level in fibroblasts, fat, and immune cells combined was computed. The *p* value from a Wilcoxon two sample test comparing epithelial to non-epithelial samples was also computed. This procedure was repeated for fibroblast, fat, and immune cells separately.

For each cell type, CpGs were ranked according to both the absolute value of the methylation difference and the *p* values. The two rankings were combined by taking the geometric mean to form a single ranking. The top 100 CpGs were selected as cell-type specific CpGs, i.e., the top CpGs were enriched for CpGs that have both a large difference in mean methylation and a small *p* value. This resulted in a total of 398 unique CpGs. The mean methylation level at each of these CpGs in each cell-type is used to create a 398 by 4 reference matrix of beta values that can be used with the EpiDISH algorithm.

A secondary immune subtype reference panel was also developed. Of the 118 immune cell samples (Supplementary Data 1), 83 had been Fluorescence-activated cell sorted (FACS sorted) into different subtypes. A reference panel was constructed using 18 B cell, 4 natural killer, 17 CD4 T cell, 16 CD8 T cell, 17 monocyte, and 7 neutrophil samples from two GEO datasets. As above, mean differential methylation and *p* values were used to rank and select 50 cell-type-specific CpGs for each cell-type. This resulted in 317 unique CpGs, that formed a 317 by 7 reference matrix which can be used with the hEpiDISH function.

A further reference panel for mammary epithelial subtypes was also constructed. Of the 46 epithelial samples described (Supplementary Data 1), 28 had been FACS sorted into different mammary epithelial subtypes. A reference panel was constructed using 10 basal (including both myoepithelial differentiated and myoepithelial progenitors), 8 luminal progenitors, and 10 mature luminal (including luminal ductal and alveolar cells) samples from two GEO datasets. As above, mean differential methylation and *p* values were used to rank and select 100 cell-type-specific CpGs for each cell-type. This resulted in 254 unique CpGs, which formed a 254 by 3 reference matrix that can be used with the hEpiDISH function.

### PRBS analysis

Genome-wide progesterone receptor interaction sites identified in the mouse uterus using ChIP-seq (GSE34927)^27^ were downloaded, and 2693 overlapping CpGs from the mouse methylation array were identified. The prbsMean quantity was defined as the mean beta value across these CpGs.

### Mitotic clock

Polycomb group target genes identified in human studies^28^ were mapped to mouse homologs (using informatics.jax.org/downloads/reports/HOM_MouseHumanSequence.rpt) resulting in 1227 mouse genes. A total of 2350 CpGs lie within 200 bp of the transcription start sites of these genes. Using the mouse methylation atlas dataset^29^ a subset of 1387 of these CpGs was identified that have less than 10% mean methylation in fetal brain, intestine, limb, and liver tissues. The mitotic clock was defined as the mean beta value across these 1387 CpGs.

### Reporting summary

Further information on research design is available in the Nature Portfolio Reporting Summary linked to this article.

## Results

### In vivo animal model

In order to investigate whether epigenetic signatures in distant “surrogate” tissues indicative of a field defect are reflective of field cancerization in the tissue at risk (the mammary gland) and whether these signatures reflect the efficacy of cancer preventive measures (i.e., mifepristone), we used a chemical-induced mouse model of breast cancer. Exposure to medroxyprogesterone acetate (MPA, subsequently referred to as “P”) and 7,12-dimethylbenzanthracene (DMBA, subsequently referred to as “D”) induced mammary tumors in female BALB/c mice, as shown previously^11^. We developed epigenetic signatures analogous to previously described human risk scores using a “Discovery set”, which consisted of 9 vehicle treated/healthy mice and 9 mice exposed to P/D, of which 8 developed a mammary tumor, as well as publicly available mouse methylation data. In this Discovery set we collected samples from normal mammary gland, mammary tumor, cervix, oviduct, blood, adipose tissue, kidney, liver, lung, and spleen from both groups. Epigenetic signatures were evaluated in the context of cancer formation in four surrogate tissues of 35 mice randomized into four groups (“Intervention set”, with and without P/D and with and without mifepristone).

### Mifepristone accumulates in breast tissue and protects against tumor development

**Fig. 2:**
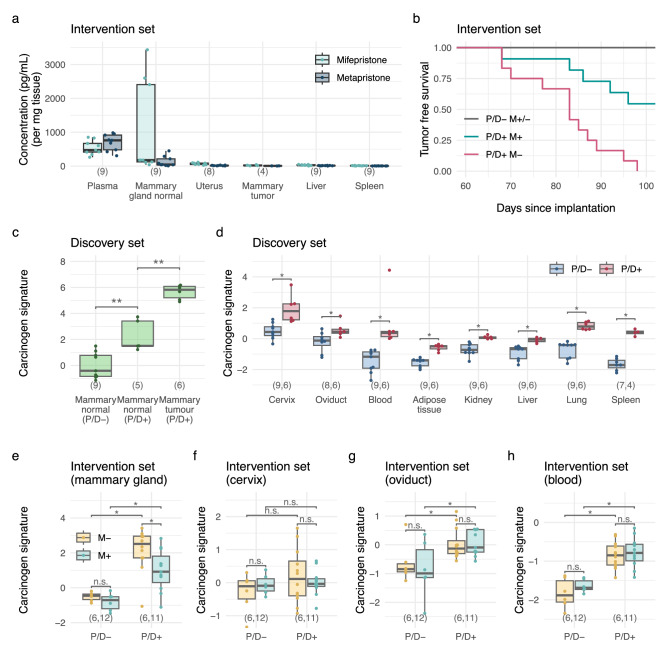
Development of a pan-tissue carcinogen signature. **a** Concentrations of mifepristone and its metabolite metapristone across different tissues in mice treated with mifepristone. **b** Kaplan-Meier curves of tumor-free survival from the Intervention set. **c**, **d** The carcinogen signature in the Discovery set. **e–h** The carcinogen signature in the Intervention set. P/D, medroxyprogesterone acetate and 7,12-dimethylbenzanthracene; M, mifepristone; P/D+ and M+ indicates the presence of P/D and M; P/D− and M− indicate the absence of P/D and M; M+/− indicates both M+ and M− experimental subgroups combined; n.s., non-significant; *p < 0.05 and **p < 0.01 based on Wilcoxon tests. P/D medroxyprogesterone acetate and 7,12-dimethylbenzanthracene, M mifepristone.

To demonstrate the tissue distribution of mifepristone and one of its active metabolites metapristone in our mouse model, blood and tissue samples from normal mammary gland, uterus, mammary tumor, liver, and spleen were collected. Our analysis revealed that mifepristone and metapristone concentrations were highest in plasma (i.e., due to the continuous release by the mifepristone-containing pellet) and normal mammary gland tissue, as expected due to their lipophilic nature (Fig. 2a).

All mice exposed to P/D in the absence of mifepristone developed mammary tumors. Mifepristone had a strong protective effect against tumor development in P/D-exposed mice when compared to mice without mifepristone treatment (*p* = 0.002; Fig. 2b).

### Carcinogen signature reflects P/D-exposure in surrogate tissues

Epigenome-wide DNAme was analyzed using the Illumina Infinium Mouse Methylation BeadChip covering approximately 287,000 CpG sites. The Discovery set was used to compare tissue samples from healthy mice to those from P/D-exposed mice to derive signatures indicative of cancer risk, analogous to previously identified epigenetic indices for humans^5,6,30,31^. Tumor samples were not included in index development as we aimed to identify differentially methylated CpG sites that change in surrogate tissues and are indicative of systemic changes rather than tumor material. For each tissue, CpG sites were ranked according to the difference in mean beta values between the two experimental subgroups. A geometric mean was used to combine tissue-specific rankings into a single ranking, thereby identifying CpGs that were hyper- or hypo-methylated across multiple tissues. A carcinogen signature was defined as the mean beta value across the top 1000 hyper-methylated CpGs minus the mean beta value across the top 1000 hypo-methylated CpGs.

Within the Discovery set, the signature was significantly elevated in normal mammary gland from P/D-exposed mice (i.e., reflective of field cancerization) and elevated further within mammary tumors (*p* < 0.01, Fig. 2c). Across all remaining tissue types, the carcinogen signature captured systemic DNAme alterations associated with carcinogenic P/D-exposure that were strikingly consistent across tissues (Fig. 2d).

A similar trend was observed in the Intervention set. Across mammary gland and field defect-indicating surrogate tissues (i.e., cervix, oviduct, and blood), the carcinogen signature was elevated in mice exposed to P/D (Fig. 2e–h). In the normal mammary gland, the signature in mice exposed to P/D was reduced by mifepristone (Fig. 2e), an effect that mirrors the overall survival outcomes of these mice (Fig. 2b). Interestingly, mifepristone also appeared to reduce the carcinogen signature in the mammary gland of healthy mice, although this was not significant (*p* = 0.24). Mifepristone did not have a significant effect on the carcinogen signature in samples from the cervix, oviduct, and blood of healthy mice (Fig. 2f–h).

### Cell-type composition changes in response to P/D-exposure and mifepristone

We estimated the cell-type composition of different tissue samples using Epigenetic Dissection of Intra-Sample-Heterogeneity (EpiDISH)^32^, an algorithm that infers the relative proportion of different cell-types using previously developed reference panels. To our knowledge, no reference panels currently exist for mice, and therefore we developed a reference panel for epithelial, fibroblast, fat, and immune cells using nine publicly available datasets on Gene Expression Omnibus (GEO; Supplementary Data 1). Secondary reference panels were developed to infer the proportion of luminal progenitor, mature luminal, and basal epithelial cell subtypes in mammary gland samples as well as B cells, NK cells, CD4+ T cells, CD8+ T cells, monocytes, neutrophils, and myeloid-derived suppressor cells (MDSC).

**Fig. 3:**
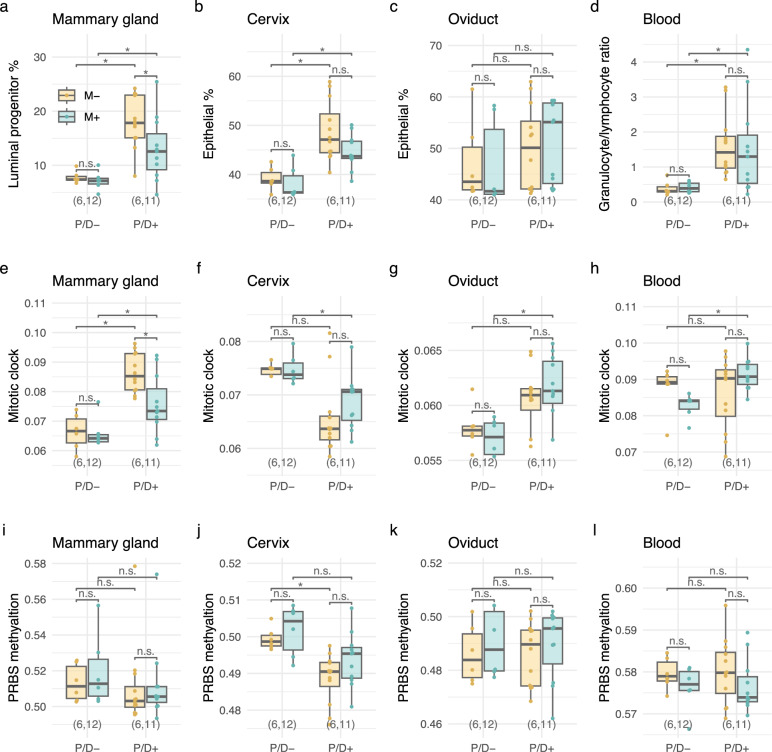
Cell-type proportions, mitotic clock, and PRBS methylation across different tissues in the Intervention set. Inferred proportions of luminal progenitor cells in mammary gland samples (**a**) epithelial cells in cervix and oviduct samples (**b**, **c**) and the ratio of granulocyte to lymphocytes in blood samples (**d**). **e–h** A mitotic clock, based on mean methylation across PCGT genes, across different tissues. **i–l** Mean progesterone receptor binding site (PRBS) methylation across different tissues. P/D, medroxyprogesterone acetate and 7,12-dimethylbenzanthracene; M mifepristone, P/D+ and M+ indicates the presence of P/D and M, P/D− and M− indicate the absence of P/D and M; *p < 0.05 and n.s., not significant, based on Wilcoxon tests. P/D medroxyprogesterone acetate and 7,12-dimethylbenzanthracene; M mifepristone.

Cell-type composition differed profoundly in normal mammary gland, cervix, oviduct, and blood samples across the different experimental subgroups (Supplementary Fig. 2). Consistent with previous findings in humans^18^ and the previously described carcinogenic mechanism of progesterone^11,14,33–35^, the proportion of luminal progenitor cells increased in normal murine mammary gland samples after exposure to P/D (again reflective of field cancerization) and was subsequently reduced by mifepristone treatment (Fig. 3a). In the cervix, the proportion of epithelial cells significantly increased in P/D-exposed mice and tened to be reduced by mifepristone (Fig. 3b). The increase in epithelial cells in oviduct samples was less distinct (Fig. 3c). The granulocyte/lymphocyte ratio in blood was increased 4.6-fold in P/D-exposed mice and not altered by mifepristone exposure (Fig. 3d).

### Mitotic clock and PRBS methylation changes in response to P/D-exposure and mifepristone

In a previous study, we observed that PCGT methylation acted like a mitotic clock^36^, and increased methylation at PCGT sites was associated with cancer^18^. We defined a mouse mitotic clock as the mean beta values across these CpGs and found that it was increased in the normal mammary gland and oviduct in P/D-exposed mice and that this effect was reduced by mifepristone in the mammary gland but not the oviduct (Fig. 3e, g). Interestingly, in the cervix we found an opposite effect, with a reduction in the mitotic clock associated with P/D-exposure; Mifepristone appeared to partially reverse this effect in the cervix, although this was not statistically significant (Fig. 3f). The observation that the mitotic clock in the blood was largely unaffected (Fig. 3h) reflects the fact that progesterone is not a substantial factor regulating the replication of peripheral blood mononuclear cells.

We identified 2693 CpGs that overlapped with PRBS and defined the PRBS methylation signature as the mean beta value across these CpG sites. We observed a significant reduction in PRBS methylation in the cervix in response to P/D-exposure, whereas PRBS methylation in the breast, oviduct, and blood was unaffected (Fig. 3i–l).

### Epigenetic signatures are predictive of survival outcomes

**Fig. 4:**
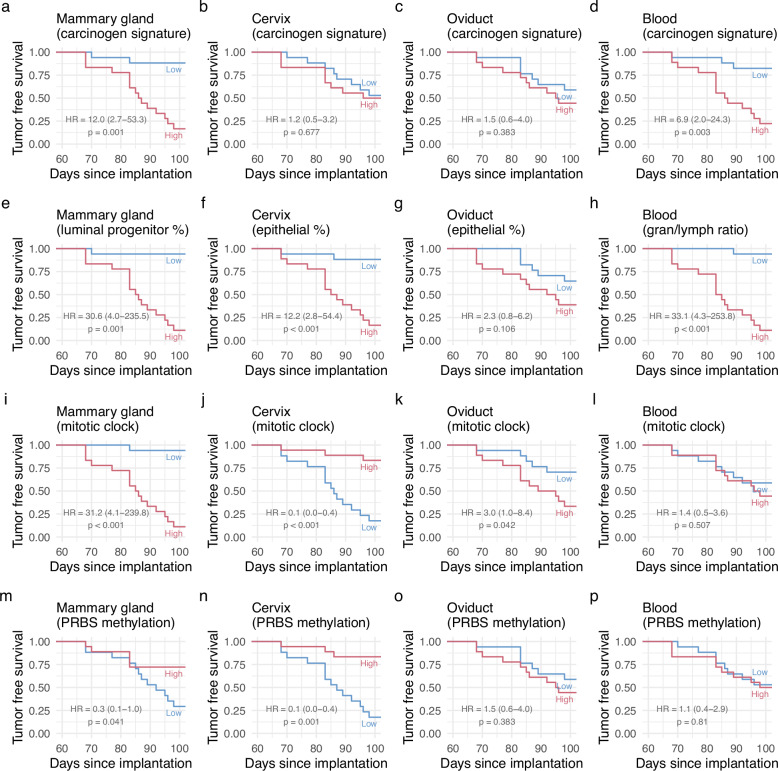
Association between tumor-free survival and the four epigenetic signatures. **a–p** Kaplan-Meier plots of tumor-free survival corresponding to the four different epigenetic signatures (rows) across four different tissues (columns) in the Intervention set. In each plot mice were split into ‘high’ or ‘low’ groups according to the median value of the respective signature. Hazard ratios (HR) with 95% confidence intervals in brackets and *p* values were calculated using a Cox proportional hazards model fitted with the respective signature included as a single covariate, comparing high versus low groups.

So far, we have only assessed the level of the signatures in the different treatment groups irrespective of cancer formation. Hence, we next analyzed the association of tumor-free survival outcomes depending on the four DNAme-based biomarkers in each of the four normal surrogate tissues (Fig. 4). In the normal mammary gland, all four biomarkers are significantly and directly associated with survival outcomes (Fig. 4a, e, i, m). In the cervix (Fig. 4b, f, j, n) epithelial cell proportion, mitotic clock, and PRBS methylation were significant predictors of survival. The association of the mitotic clock with cancer risk was opposite in the cervix (Fig. 4j) when compared to the mammary gland (Fig. 4i). In the oviduct (Fig. 4c, g, k, o), only the mitotic clock (Fig. 4k) is a significant, albeit modest, predictor of cancer-free survival. In blood (Fig. 4d, h, l, p), the carcinogen signature (Fig. 4d) and the granulocyte/lymphocyte ratio (Fig. 4h) are significant predictors of outcome.

## Discussion

Here we have demonstrated that in mice that end up developing mammary gland cancer, strong effects of field cancerization are observed in the normal mammary gland. Beside a signature solely reflecting the carcinogen exposure, all three other signatures indicative of specific mechanisms are altered in normal mammary glands of mice that developed a cancer: A high proportion of luminal progenitor cells, which are known to be the cells of origin of progesterone-mediated breast cancers^33,35^, a high replicative age indicated by the mitotic clock and consistent with the high proliferative activity in the carcinogen-exposed mammary gland and a low level of methylation of PRBS support the view that high/continuous progesterone levels reduce stochastic accumulation of methylation at PRBS sites, thereby enabling progesterone to continue exerting its activity.

In humans, multiple breast biopsies to assess the level of field cancerization over time to guide preventive measures are not practical and feasible for patients. Hence, biomarkers in easy to access tissue that reliably reflect field cancerization and thereby act as a field defect indicator are urgently needed. The data presented here indicate that cervical samples are the most effective surrogate field defect indicators. Both parameters which are reflective of progesterone’s role in breast carcinogenesis - i.e., mitotic activity and methylation at PRBS - are mirrored in cervical cells. Whereas low PRBS methylation is present in both the mammary gland and the cervix of mice with cancer, a high replicative age in the normal mammary gland and a low replicative age in the cervix are indicative of cancer.

Field cancerization, which is the replacement of a normal cell population by a cancer-primed cell population that may show no morphological change, is now recognized to underlie the development of many types of cancer^2^.

For the first time, we have demonstrated that epigenetic signatures assessed in normal mammary glands are predictive of cancer formation in one or more of the remaining mammary glands of the same mouse. Moreover, epigenetic signatures in easy to access cervicovaginal tissue very closely mirror the effects of both the carcinogen exposure and the preventive effects of mifepristone, thus facilitating the prediction of the cancer-preventive response to mifepristone with a high degree of accuracy. These findings are aligned with prior evidence demonstrating that epigenetic signatures in cervical smear samples can identify women who will develop breast cancer with the worst prognoses^5^ and with observations of a deceleration in the relative epithelial age in the cervical smear samples of premenopausal women with breast cancer and in normal breast tissue from *BRCA1* mutation carriers which is reversed by antiprogestins^37^. This phenomenon is consistent with the fact that the mitotic clock captures tissue-specific biological effects - i.e., a progesterone-mediated increase of stem/progenitor proliferation in the mammary gland^11,38^ and a progesterone-mediated decrease in the cervix^39^. This again highlights the fact that these signatures are tissue-specific read-outs of effects triggered by the carcinogen and modulated by the preventive drug. Most importantly, even though distant surrogate tissue (i.e., the cervix) shows an opposite effect compared to the tissue at risk (i.e., the mammary gland) the information obtained reflects cancer-predisposition and the efficacy of preventive measures.

Surprisingly, although progesterone receptors are expressed in the oviduct as well and have been implicated in the carcinogenesis and mifepristone-mediated prevention of cancers in this organ^16^, compared to the cervix, the mitotic clock in the oviduct is substantially less informative in terms of mammary gland cancer and PRBS methylation is not at all an indicator of cancer formation in this tissue.

Consistent with the progesterone-inert status of blood cells, neither the mitotic clock nor PRBS were suitable field defect indicators in blood cells. Whether the high granulocyte/lymphocyte ratio associated with mammary gland cancer is indicative of cancer risk or merely reflective of an immune response to the cancer cannot be assessed based on the experimental setup we have chosen.

We have conclusively demonstrated that epigenetic signatures define field cancerization and when assessed in field defect-indicating distant organs - mainly the cervix - can indicate cancer formation. But it remains unclear as to how long in advance of cancer formation, field cancerization develops, whether field defect indicators can predict the future cancer risk in women and at what “risk level” mifepristone can reduce field defect indicating signatures and whether this enables cancer precision prevention. In order to address this, a longitudinal set-up including sequential samples from both the mammary gland and the cervix would be required. The fact that the cervix is not accessible in a live mouse makes this setting impossible. In addition, future studies could investigate the long-term dynamics of the field defect after mifepristone treatment is stopped.

Overall, our data add to the portfolio of evidence calling for a clinical trial in high-risk women to assess whether mifepristone can reduce the risk of breast cancers with the worst prognoses, using epigenetic signatures in cervical smear samples as field defect indicators to monitor the preventive effects of treatment.

## Supplementary information

Supplementary Information

Description of Additional Supplementary Files

Supplementary Data 1

Supplementary Data 2

Reporting summary

## Data Availability

The mouse DNA methylation data are deposited in GEO (submission number GSE236260). The data used to generate all plots (source data) are available in Supplementary Data 2.

## Code availability

All analyses were run on the open-source software R, version 4.4.1, leveraging standard libraries. Code for the carcinogen signature is deposited on Zenodo^26^. This script is available under a Gnu General Public Licence.
