# Supplementary material for: Epigenetic signatures in surrogate tissues are able to assess cancer risk and indicate the efficacy of preventive measures
Source: Commun Med (Lond). 2025 Apr 2;5:97. doi: 10.1038/s43856-025-00779-w (PMC11965489; doi:10.1038/s43856-025-00779-w)
Supplement: Supplementary file 1 — Supplementary Information [file 43856_2025_779_MOESM1_ESM.pdf]

Supplementary Materials for

Epigenetic signatures in surrogate tissues are able to assess cancer risk and indicate the efficacy of preventive measures

James E. Barrett†, Chiara Herzog†, Sepideh Aminzadeh-Gohari†, Elisa Redl, Isma Ishaq Parveen, Julia Rothärmel, Julia Tevini, Daniela D. Weber, Luca Catalano, Victoria Stefan, Thomas K. Felder, Peter Obrist, Twana Alkasalias, Kristina Gemzell-Danielsson, Roland Lang, Barbara Kofler, Martin Widschwendter*

† These authors contributed equally to this work.

*Corresponding author. E-Mail: Martin.Widschwendter@uibk.ac.at

The PDF file includes:

Supplementary Figs. 1 to 2.
References.

Other Supplementary Materials for this manuscript include the following:

Supplementary Data 1 – separate xlsx file.
Supplementary Data 2 – separate csv file.

Supplementary Figures

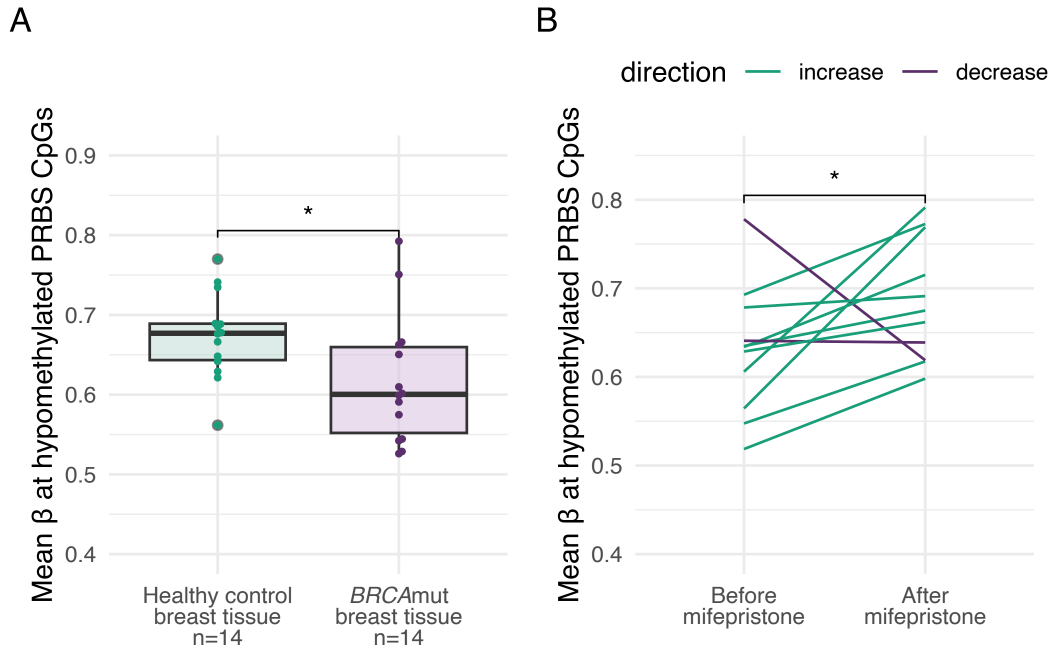

Supplementary Fig. 1 | a Mean PRBS methylation in human breast tissue samples from healthy women compared to breast tissue samples from *BRCA1* mutation carriers. * p <0.05 in Wilcoxon test (note, PRBS-definition in this context and panel has been published previously [1] and is included here only for consistency and in order to compare the dynamical changes in the context of mifepristone exposure shown in panel B). **b** Mean PRBS methylation in human breast tissue samples from healthy women with a *BRCA* mutation before and after mifepristone treatment. ** p <0.01 in paired Wilcoxon (signed-rank) test.

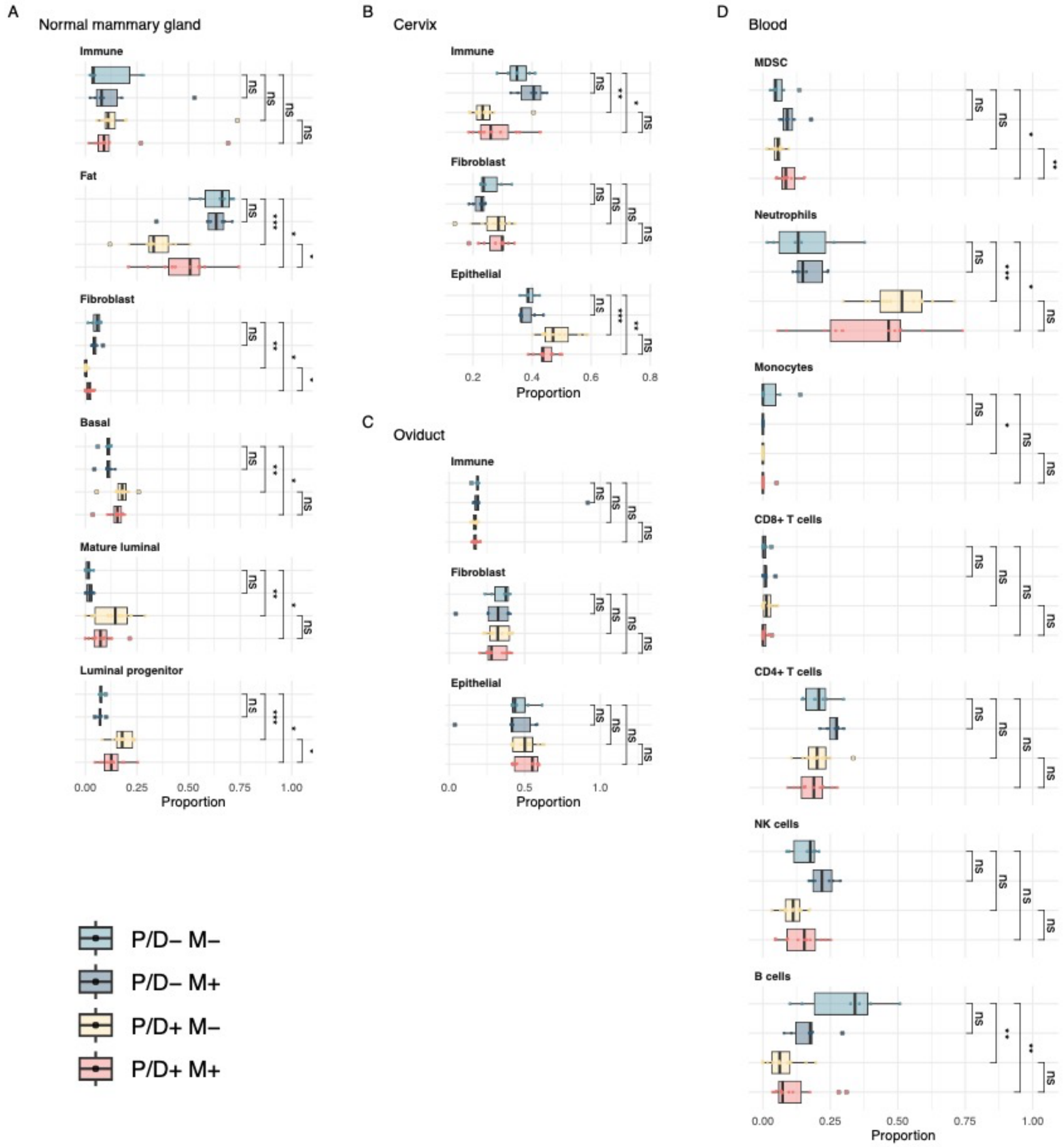

Supplementary Fig. 2 | Inferred cell-type proportions in the Intervention set. a Normal mammary gland. **b** Cervix. **c** Oviduct. **d** Blood. P/D, medroxyprogesterone acetate and 7,12-dimethylbenzanthracene; M, mifepristone; P/D+ and M+ indicates the presence of P/D and M, P/D- and M- indicates the absence of P/D and M. P/D, medroxyprogesterone acetate and 7,12-dimethylbenzanthracene; M, mifepristone.

Supplementary Data

Supplementary Data 1.

Sources of data to generate reference panels for cell subtypes using publicly available datasets on Gene Expression Omnibus (see xlsx file).

Supplementary Data 2.
Numerical data used to generate plots in the main manuscript.

References

1 Barrett, J. E. *et al.* The WID-BC-index identifies women with primary poor prognostic breast cancer based on DNA methylation in cervical samples. *Nat Commun* **13**, 449, doi:10.1038/s41467-021-27918-w (2022).
