# Supplementary material for: Epigenetic signatures in surrogate tissues are able to assess cancer risk and indicate the efficacy of preventive measures
Source: Commun Med (Lond). 2025 Apr 2;5:97. doi: 10.1038/s43856-025-00779-w (PMC11965489; doi:10.1038/s43856-025-00779-w)
Supplement: Supplementary file 2 — Description of Additional Supplementary Files [file 43856_2025_779_MOESM2_ESM.pdf]

Description of Additional Supplementary Files

File name: Supplementary Data 1.

File description: Sources of data to generate reference panels for cell subtypes using publicly available datasets on Gene Expression Omnibus (see xlsx file).

File name: Supplementary Data 2.

File description: Numerical data used to generate plots in the main manuscript.
