# Supplementary material for: Epigenetic signatures in surrogate tissues are able to assess cancer risk and indicate the efficacy of preventive measures
Source: Commun Med (Lond). 2025 Apr 2;5:97. doi: 10.1038/s43856-025-00779-w (PMC11965489; doi:10.1038/s43856-025-00779-w)
Supplement: Supplementary file 5 — Reporting summary [file 43856_2025_779_MOESM5_ESM.pdf]

nature portfolio

Corresponding author(s): Martin Widschwendter

Last updated by author(s): Apr 15, 2024

Reporting Summary

Nature Portfolio wishes to improve the reproducibility of the work that we publish. This form provides structure for consistency and transparency in reporting. For further information on Nature Portfolio policies, see our Editorial Policies and the Editorial Policy Checklist.

Statistics

For all statistical analyses, confirm that the following items are present in the figure legend, table legend, main text, or Methods section.

n/a	Confirmed	
☐	☒	The exact sample size (*n*) for each experimental group/condition, given as a discrete number and unit of measurement
☐	☒	A statement on whether measurements were taken from distinct samples or whether the same sample was measured repeatedly
☐	☒	The statistical test(s) used AND whether they are one- or two-sided<br>*Only common tests should be described solely by name; describe more complex techniques in the Methods section.*
☐	☒	A description of all covariates tested
☐	☒	A description of any assumptions or corrections, such as tests of normality and adjustment for multiple comparisons
☐	☒	A full description of the statistical parameters including central tendency (e.g. means) or other basic estimates (e.g. regression coefficient) AND variation (e.g. standard deviation) or associated estimates of uncertainty (e.g. confidence intervals)
☐	☒	For null hypothesis testing, the test statistic (e.g. *F*, *t*, *r*) with confidence intervals, effect sizes, degrees of freedom and *P* value noted<br>*Give P values as exact values whenever suitable.*
☐	☒	For Bayesian analysis, information on the choice of priors and Markov chain Monte Carlo settings
☐	☒	For hierarchical and complex designs, identification of the appropriate level for tests and full reporting of outcomes
☐	☒	Estimates of effect sizes (e.g. Cohen's *d*, Pearson's *r*), indicating how they were calculated

Our web collection on statistics for biologists contains articles on many of the points above.

Software and code

Policy information about availability of computer code

Data collection	No software used
Data analysis	Methylation analysis was conducted using a custom pipeline (eutopsQC). Briefly, raw methylation microarray data were loaded using the R package minfi, version 1.46, with minor modifications to allow for annotation of the mouse array, which is not by default included in the minfi package. For this, we generated annotation (https://www.github.com/chiaraherzog/IlluminaMouseMethylationanno.12.v1.mm10) and manifest (https://www.github.com/chiaraherzog/IlluminaMouseMethylationmanifest) R packages based on the array information provided by Illumina. Any samples with median methylated and unmethylated intensities <9.5 were removed. Any probes with a detection p-value >0.01 were regarded as failed. Any samples with >10% failed probes, and any probes with a >10% failure rate were removed from the dataset. A total of six samples were removed (one oviduct and two spleen samples from healthy mice in the Discovery set, and one mammary gland and two spleen samples from P/D-exposed mice in the Discovery set).<br>Background intensity correction and dye bias correction was performed using the minfi single sample preprocessNoob function. Probe bias correction was performed using the beta mixture quantile normalization (BMIQ) algorithm in the ChAMP package, version 2.30.0, with a modification to allow for processing of the mouse array. The top 1,000 most variable probes (ranked by standard deviation) were used in a principal component analysis. Correlation or Kruskal-Wallis tests were performed in order to identify any anomalous associations between plate, sentrix position, date of array processing, date of DNA isolation, sampling date, DNA concentration, and the top ten principal components. No issues were identified.

For manuscripts utilizing custom algorithms or software that are central to the research but not yet described in published literature, software must be made available to editors and reviewers. We strongly encourage code deposition in a community repository (e.g. GitHub). See the Nature Portfolio guidelines for submitting code & software for further information.

Data

Policy information about availability of data

All manuscripts must include a data availability statement. This statement should provide the following information, where applicable:

- Accession codes, unique identifiers, or web links for publicly available datasets
- A description of any restrictions on data availability
- For clinical datasets or third party data, please ensure that the statement adheres to our policy

Data will be uploaded to GEO. Accession number will be provided prior to publication.

Research involving human participants, their data, or biological material

Policy information about studies with human participants or human data. See also policy information about sex, gender (identity/presentation), and sexual orientation and race, ethnicity and racism.

Reporting on sex and gender	n/a
Reporting on race, ethnicity, or other socially relevant groupings	n/a
Population characteristics	n/a
Recruitment	n/a
Ethics oversight	n/a

Note that full information on the approval of the study protocol must also be provided in the manuscript.

Field-specific reporting

Please select the one below that is the best fit for your research. If you are not sure, read the appropriate sections before making your selection.

☒ Life sciences ☐ Behavioural & social sciences ☐ Ecological, evolutionary & environmental sciences

For a reference copy of the document with all sections, see nature.com/documents/nr-reporting-summary-flat.pdf

Life sciences study design

All studies must disclose on these points even when the disclosure is negative.

Sample size	n=53 mice in total
Data exclusions	none
Replication	n=18 mice were used as a "discovery set" to establish various signatures and n=35 mice were used as a "validation set" to assess the performance of the signatures
Randomization	mice were randomised into different experimental groups (either with/without carcinogen exposure, and with/without mifepristone treatment)
Blinding	Blinding was not possible

Reporting for specific materials, systems and methods

We require information from authors about some types of materials, experimental systems and methods used in many studies. Here, indicate whether each material, system or method listed is relevant to your study. If you are not sure if a list item applies to your research, read the appropriate section before selecting a response.

Materials & experimental systems

n/a	Involved in the study
☒	☐ Antibodies
☒	☐ Eukaryotic cell lines
☒	☐ Palaeontology and archaeology
☐	☒ Animals and other organisms
☒	☐ Clinical data
☒	☐ Dual use research of concern
☒	☐ Plants

Methods

n/a	Involved in the study
☒	☐ ChIP-seq
☒	☐ Flow cytometry
☒	☐ MRI-based neuroimaging

Animals and other research organisms

Policy information about studies involving animals; ARRIVE guidelines recommended for reporting animal research, and Sex and Gender in Research

Laboratory animals	female BALB/c mice (Charles River)
Wild animals	n/a
Reporting on sex	All female
Field-collected samples	n/a
Ethics oversight	Animal experiments were performed at the animal facility of the Paracelsus Medical University Salzburg in accordance with the Austrian federal ministry of education, science, and research (BMBWF), study approval No. 2021-0.236.530.

Note that full information on the approval of the study protocol must also be provided in the manuscript.

Plants

Seed stocks	n/a
Novel plant genotypes	n/a
Authentication	n/a
